# Supplementary figures and images for: Recycling of cell surface membrane proteins from yeast endosomes is regulated by ubiquitinated Ist1
Source: J Cell Biol. 2022 Sep 20;221(11):e202109137. doi: 10.1083/jcb.202109137 (PMC9491851; doi:10.1083/jcb.202109137)

Figure 2C

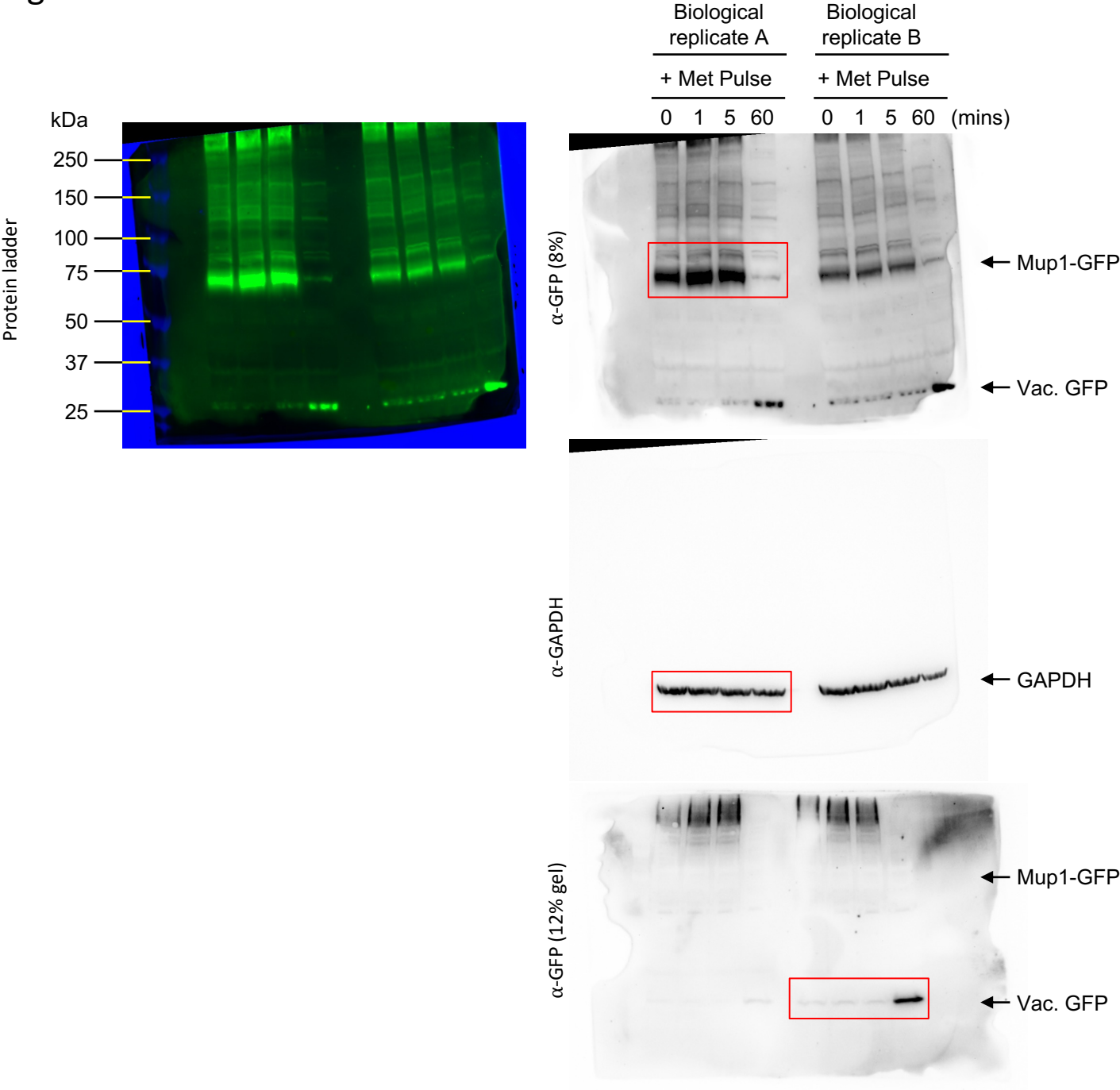

Supplement: SourceData F2 — contains original blots for Fig. 2. [file JCB_202109137_SourceDataF2.pdf]

Figure 8B  
IST1<sup>WT</sup>

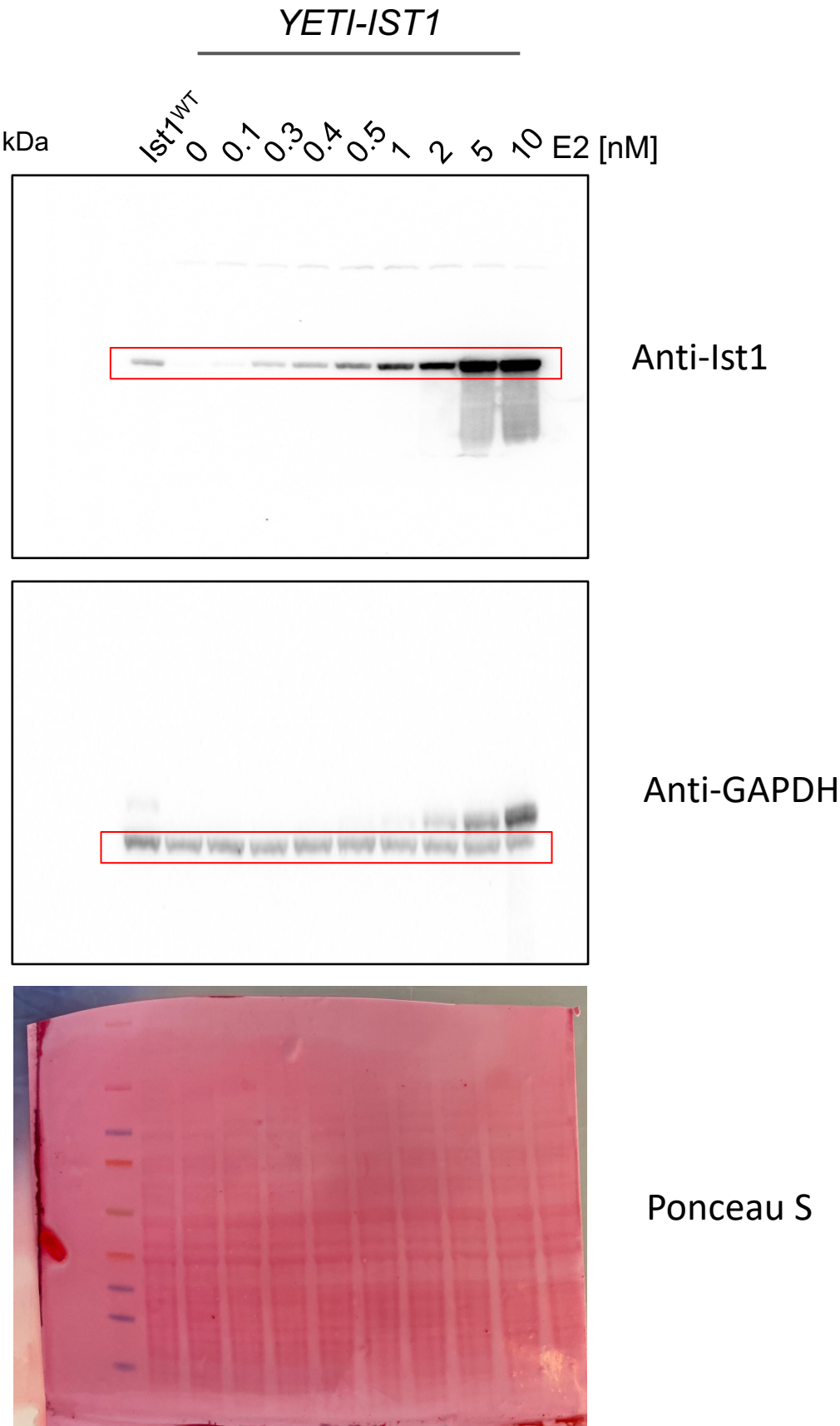

Figure 8B  
IST<sup>KR</sup>

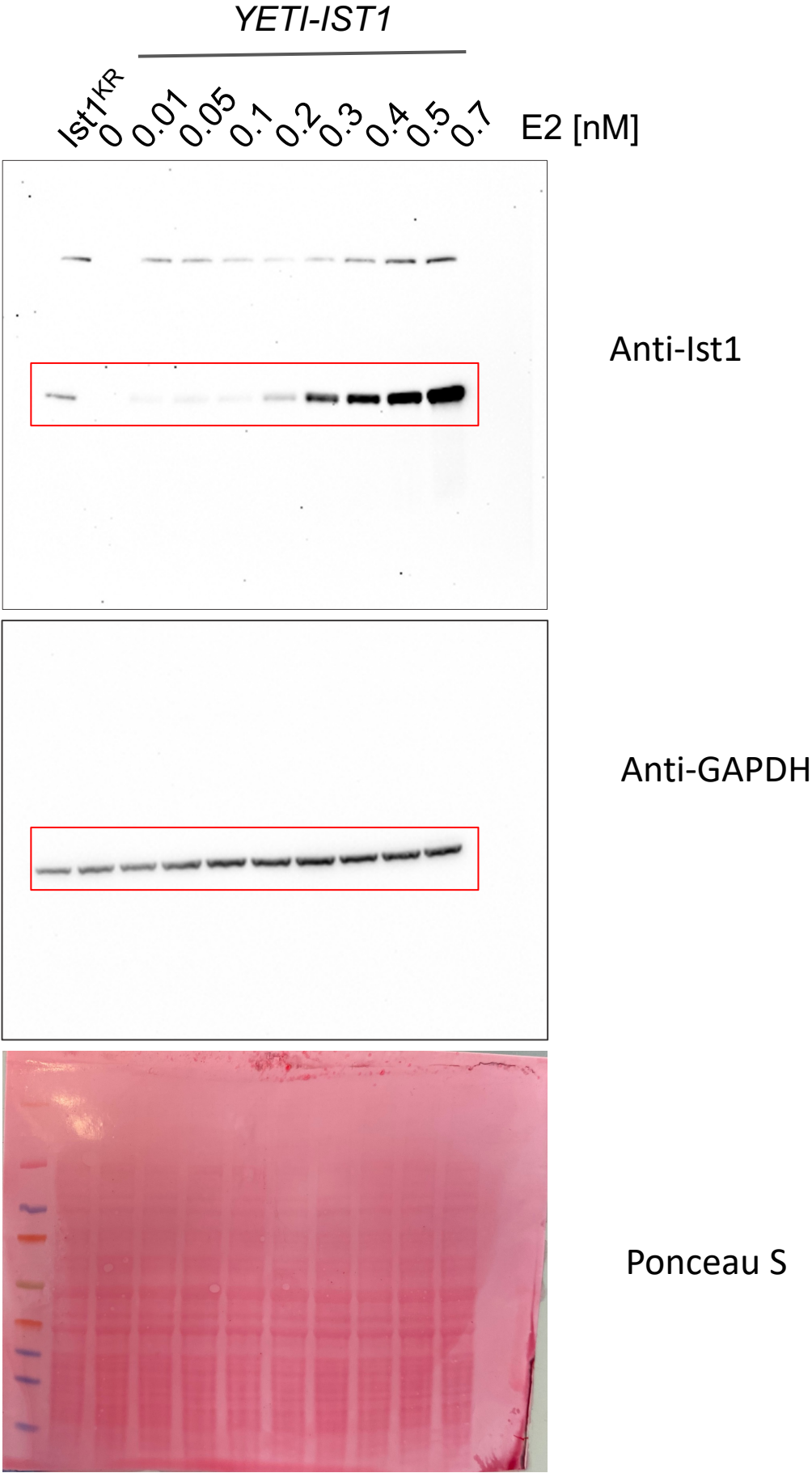

Supplement: SourceData F8 — contains original blots for Fig. 8. [file JCB_202109137_SourceDataF8.pdf]
