# Supplementary material for: Recycling of cell surface membrane proteins from yeast endosomes is regulated by ubiquitinated Ist1
Source: J Cell Biol. 2022 Sep 20;221(11):e202109137. doi: 10.1083/jcb.202109137 (PMC9491851; doi:10.1083/jcb.202109137)
Supplement: SourceData F6 — contains original blots for Fig. 6. [file JCB_202109137_SourceDataF6.pdf]

Figure 6C

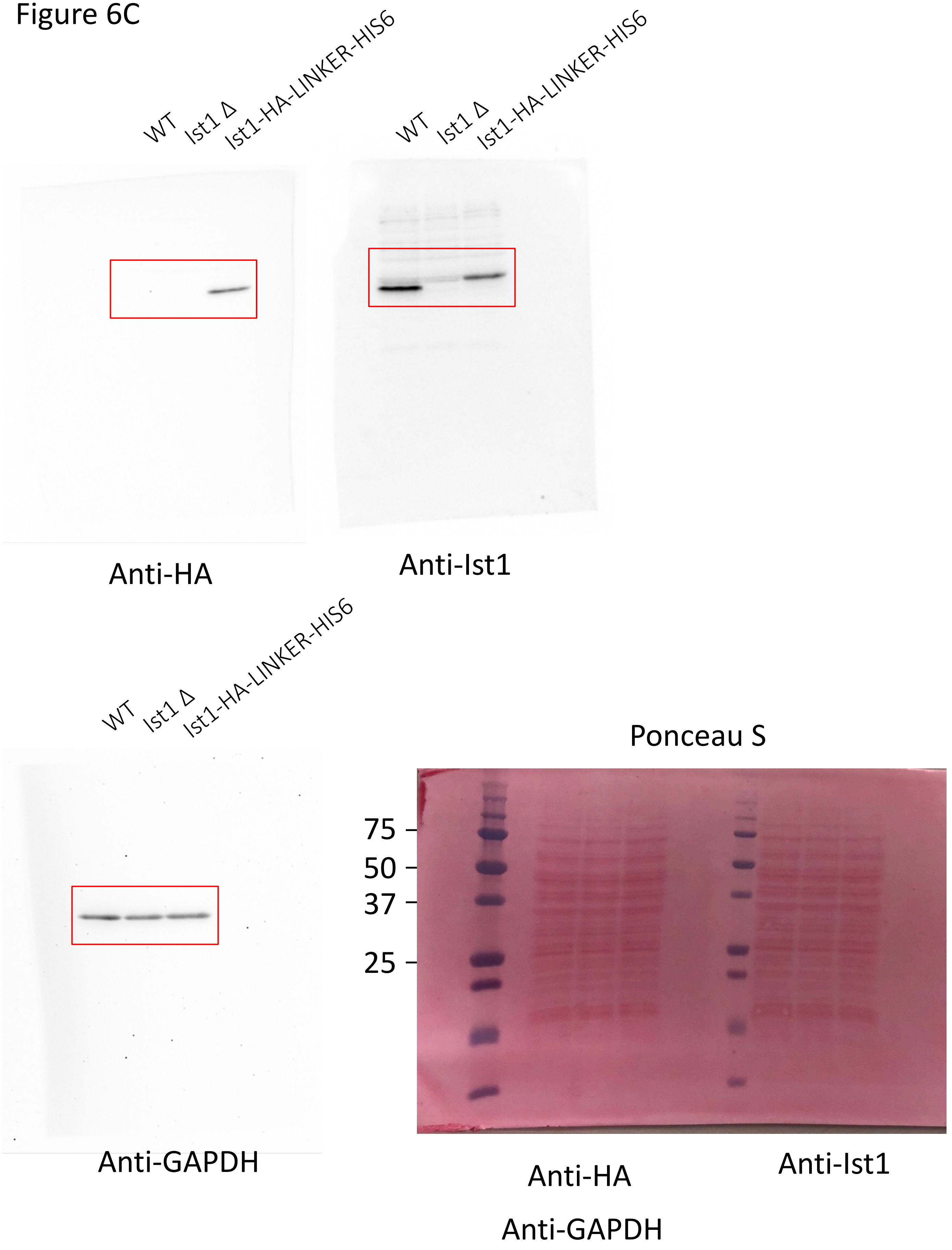
WT
Ist1 Δ
Ist1-HA-LINKER-HIS6
WT
Ist1 Δ
Ist1-HA-LINKER-HIS6
Anti-HA
Anti-Ist1
WT
Ist1 Δ
Ist1-HA-LINKER-HIS6
Ponceau S
75
50
37
25
Anti-GAPDH
Anti-HA
Anti-GAPDH
Anti-Ist1

Figure 6D

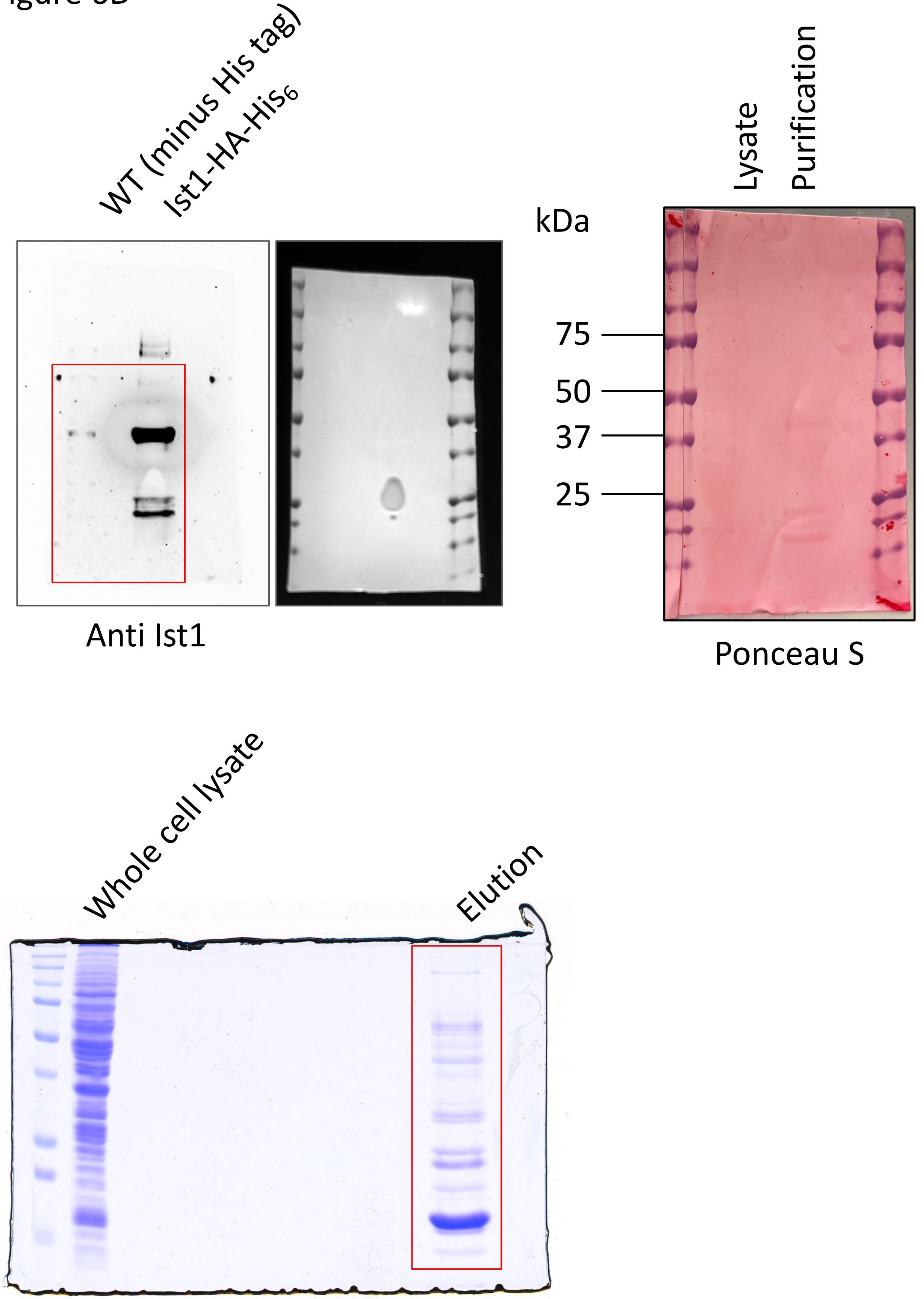

WT (minus His tag)
Ist1-HA-His6
Anti Ist1
Lysate
Purification
kDa
75
50
37
25
Ponceau S
Whole cell lysate
Elution

Figure 6H

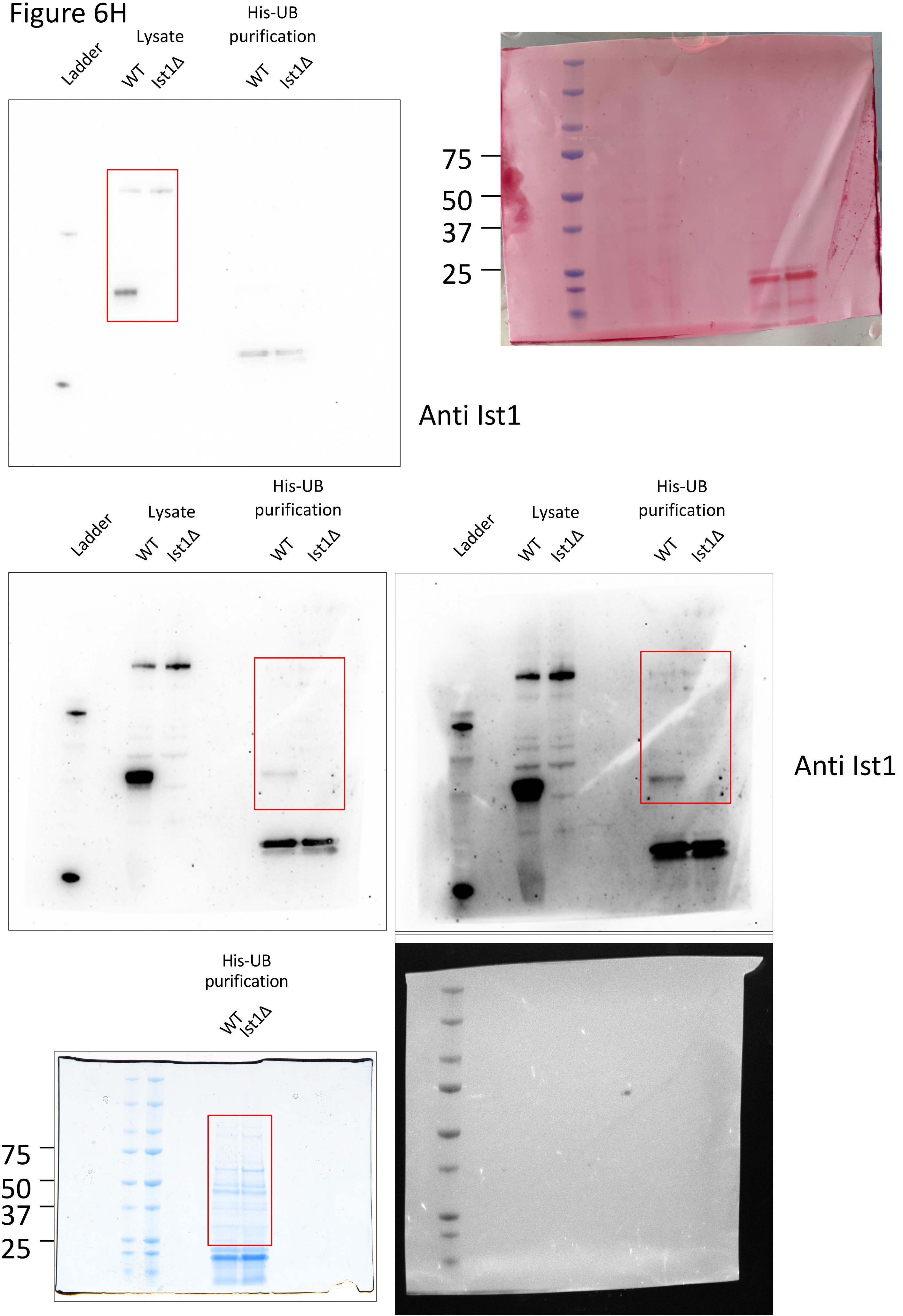

His-UB
purification
Lysate
Ladder
WT
Ist1Δ
WT
Ist1Δ
75
50
37
25
Anti Ist1
His-UB
purification
Lysate
Ladder
WT
Ist1Δ
WT
Ist1Δ
His-UB
purification
Lysate
Ladder
WT
Ist1Δ
WT
Ist1Δ
Anti Ist1
His-UB
purification
WT
Ist1Δ
75
50
37
25
