# Supplementary material for: Recycling of cell surface membrane proteins from yeast endosomes is regulated by ubiquitinated Ist1
Source: J Cell Biol. 2022 Sep 20;221(11):e202109137. doi: 10.1083/jcb.202109137 (PMC9491851; doi:10.1083/jcb.202109137)

Figure 7A

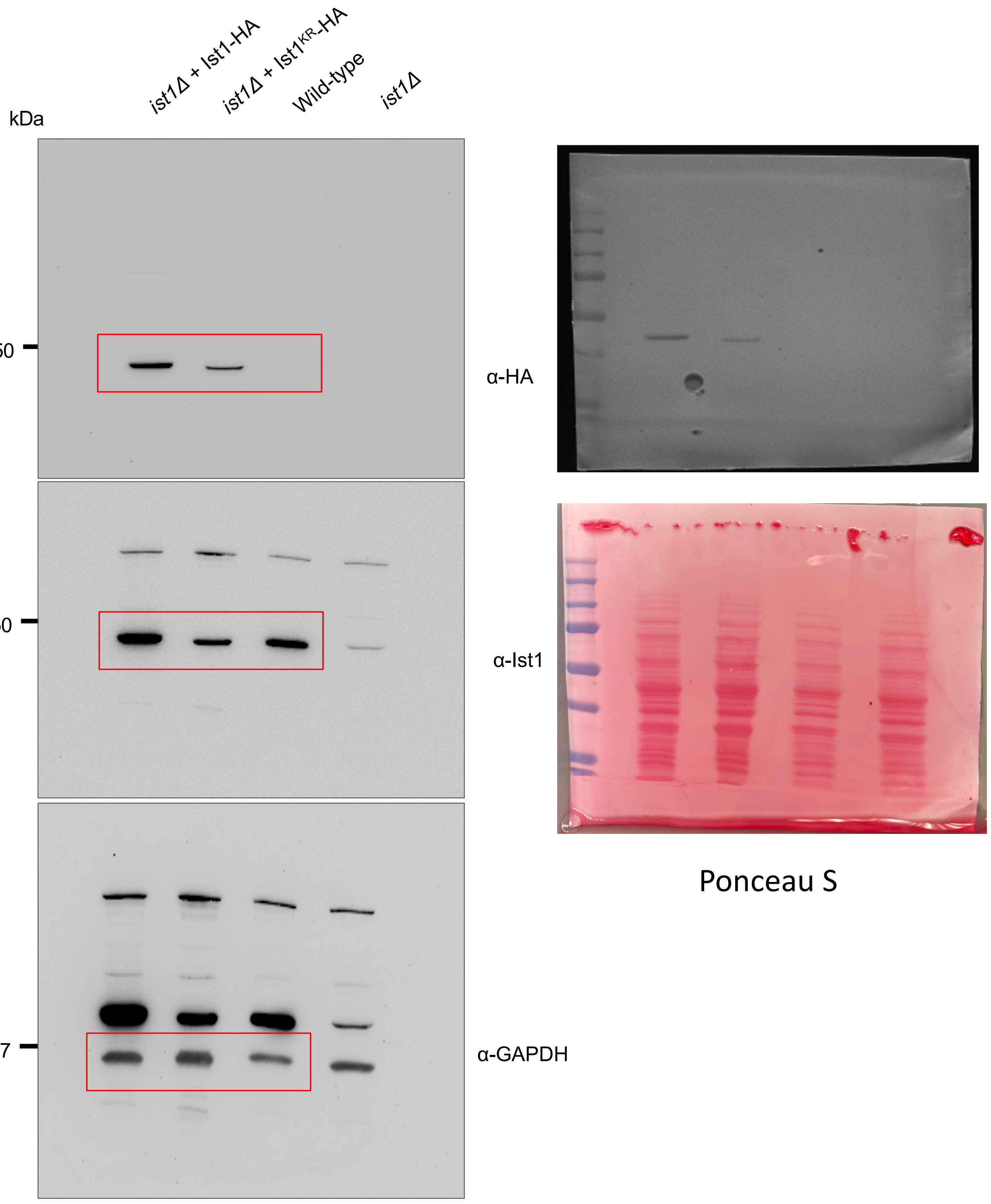

ist1Δ + Ist1-HA
ist1Δ + Ist1KR-HA
Wild-type
ist1Δ
kDa
α-HA
α-Ist1
α-GAPDH
Ponceau S

Figure 7B

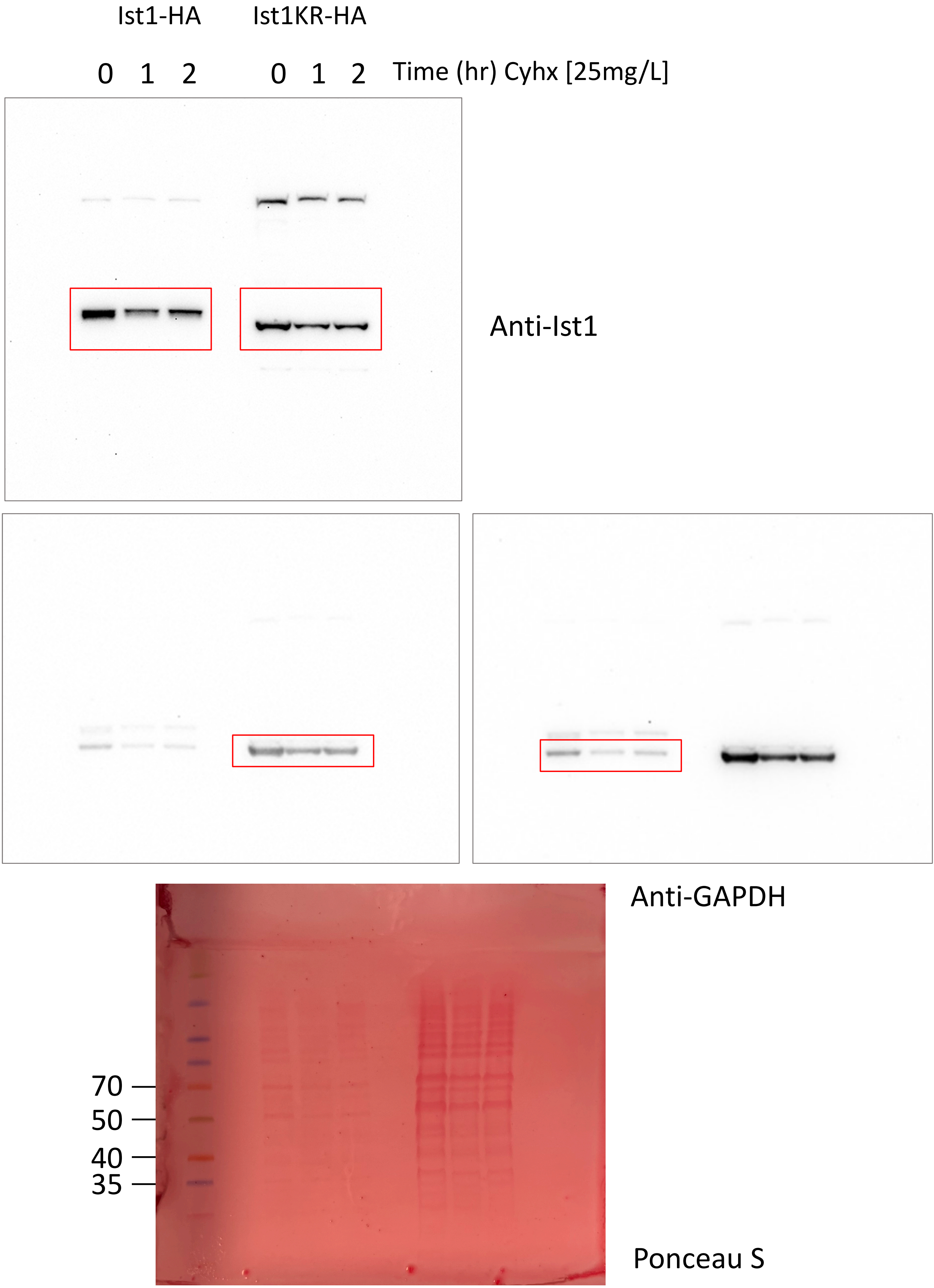

Ist1-HA
Ist1KR-HA
0 1 2
0 1 2
Time (hr) Cyhx [25mg/L]
Anti-Ist1
Anti-GAPDH
70
50
40
35
Ponceau S

Figure 7H

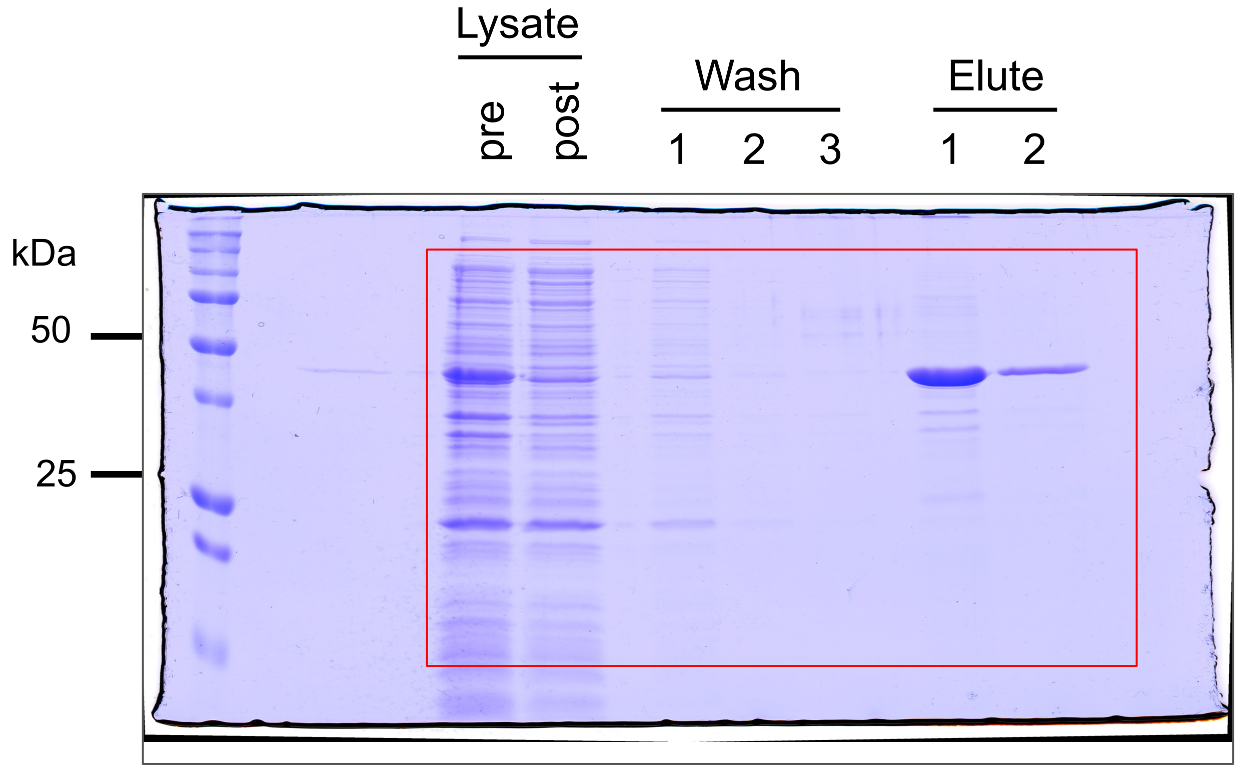

Figure 7I

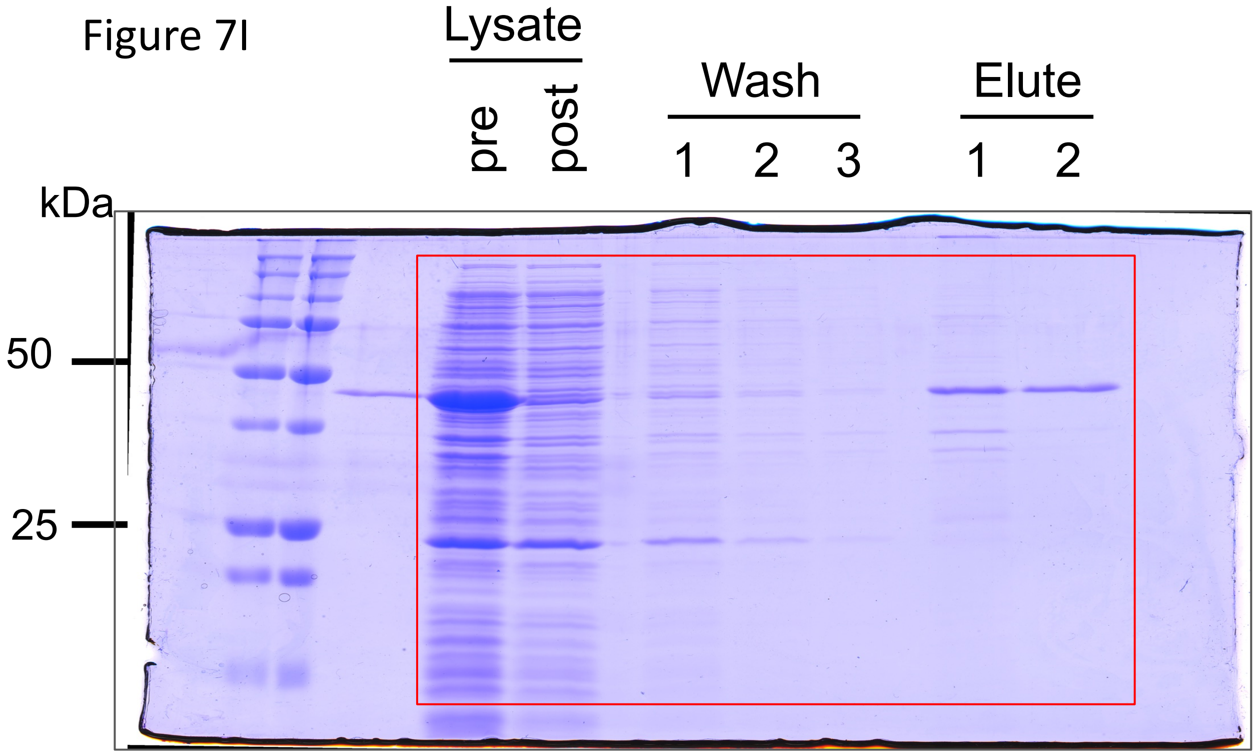

Supplement: SourceData F7 — contains original blots for Fig. 7. [file JCB_202109137_SourceDataF7.pdf]
